# Evaluation of Silibinin Effect on U-CH2 and MCF-7 Cell Lines through xCELLigence System

**DOI:** 10.34172/apb.2023.002

**Published:** 2021-10-26

**Authors:** Zohreh Jahanafrooz, Beate Rinner

**Affiliations:** ^1^Department of Biology, Faculty of Sciences, University of Maragheh, Maragheh, Iran; ^2^Division of Biomedical Research, Medical University Graz, Graz, Austria

## Dear Editor,

**Figure 1:**
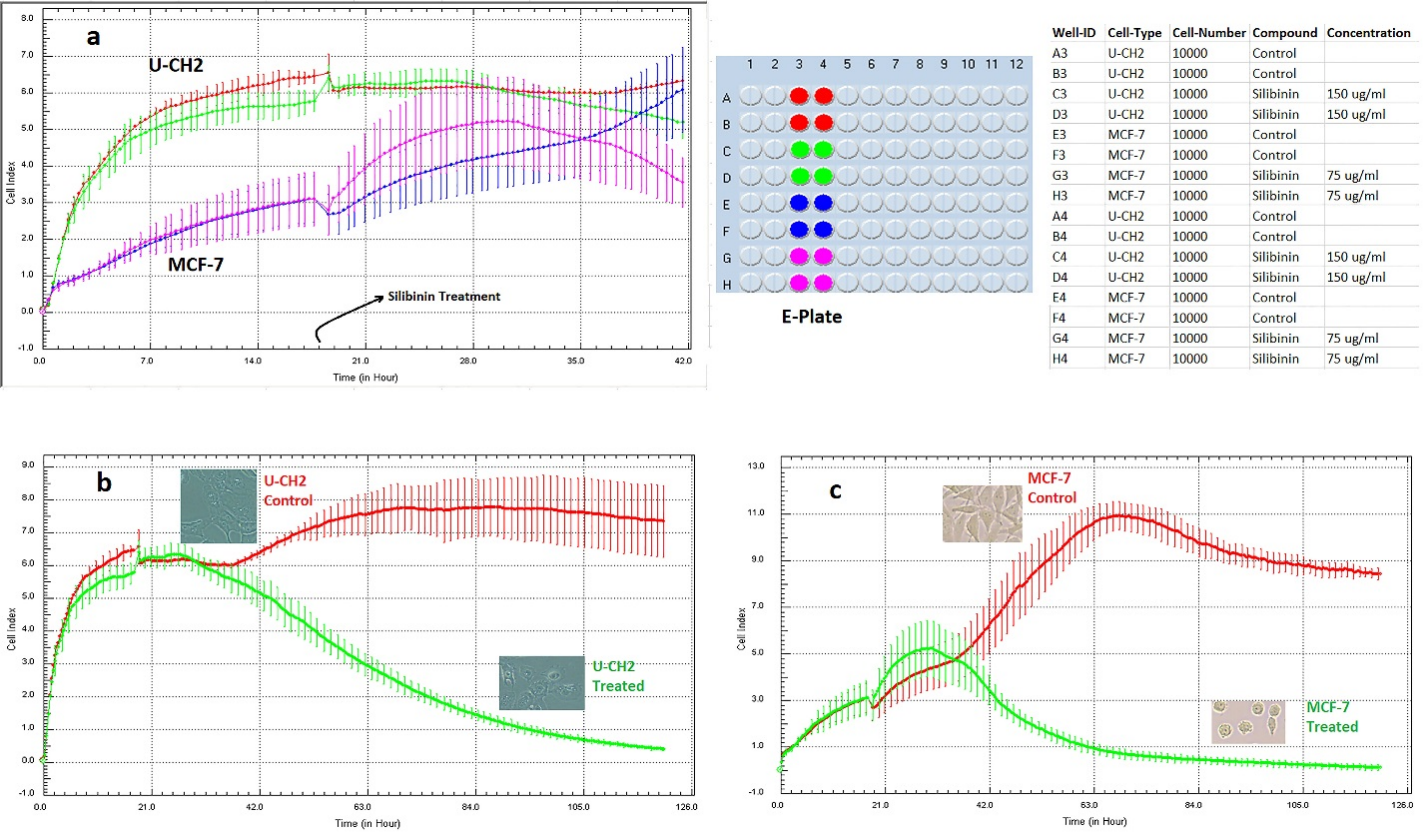
Real-time viability assessment of MCF-7 and U-CH2 cell lines under silibinin treatment. **a**: The whole-cell index of U-CH2 (red and green graphs) is higher than the MCF-7 (blue and pink graphs) because of the larger cell size of U-CH2. Silibinin treatment was performed at the 18^th^ hour after cell seeding (in about 60% confluence). Silibinin effect began at least 10 hours later. As shown here, silibinin reduced the cell index of MCF-7 (i.e., 2.5) more than (i.e., 1.1) U-CH2 cells. Vertical lines show standard deviations. E-plate and characteristic of its wells depicted on the top-right. **b**: xCELLigence assessment of U-CH2 cells under silibinin treatment until 100^th^ hour. The reduction of the cell index is 7. **c**: xCELLigence assessment of MCF-7 cells under silibinin treatment until 100^th^ hour. The reduction of the cell index is 8.2. **b** and **c**: control groups are in red color, and treated groups are in the green graph. According to phase contrast microscopy (after 48h treatment), silibinin caused condensation of U-CH2 cells and reduction in cell proliferation and viability of MCF-7 cells. Dead and detached MCF-7 cells had round shapes.

 Silibinin is a polyphenolic flavonolignan isolated from milk thistle and has diverse effects in different cancer cell lines. Silibinin showed a significant cytotoxic effect on various human cancer cell lines such as MCF-7, T47D, DU145, and PC3 cells and less cytotoxic effect on some other cancer cell lines such as MUG-CC1, MUG-Chor1, and U-CH2.^1-4^ Here, we investigated and compared proliferation and viability of U-CH2 (human sacral chordoma cell line) and MCF-7 (human breast adenocarcinoma cell line) cells under silibinin treatment through xCELLigence system, as a one accurate anticancer drug screening system. This system is dynamic monitoring of cellular phenotypic changes in real-time using impedance as readout. The system measures electrical impedance across interdigitated micro-electrodes integrated on the bottom of E-plates displayed as the cell index (CI) value. CI value provides quantitative information about the biological status of the cells. Different cellular changes (e.g., cell growth, cell proliferation, cell morphology, cell migration, and cell death) can alter CI in xCELLigence assessment.^5^ Real-time viability assessment of MCF-7 cell lines under silibinin treatment confirmed our previous results. Both proliferation and viability or metabolic activity of MCF-7 cells are influenced under silibinin treatment. This is in accordance with both phase contrast microscopy and our MTT and apoptosis results previously reported.^1^ Interestingly, at first exposure hours, silibinin caused an increase in the CI of MCF-7 cells, but it was not a significant one; it can result from the antioxidant properties of silibinin or other imposed alterations of culture media (such as pH change) after silibinin administration at first exposure hours, that needs other experiments to clarify exactly.^6^ As shown by phase contrast microscopy in Figure 1, silibinin mainly influenced the morphology of U-CH2 cells at 48 hours after silibinin treatment and caused a slight decrease in CI. According to our previous MTS and apoptosis results, silibinin induced low cytotoxicity on U-CH2 viability and proliferation at 48 hours^2^; therefore, silibinin affected the morphology of U-CH2 cells and their attachment to the surface at first two days. U-CH2 cells reacted more slowly to silibinin; it can be assumed that the plasma membrane of U-CH2 cells are less permeable to silibinin because, according to our previous report,^2^ at higher concentrations of silibinin (i.e., more than 300 ug/mL in which normal cells are also affected) U-CH2 cells showed more sensitivity. Another notable issue is that silibinin’s effect on cell proliferation and viability did not start immediately, and at least it takes about 10 hours to be effective (Figure 1). Together, real-time viability assessment is a more sensitive and valuable approach for the evaluation of anticancer agents. According to this xCELLigence assessment, silibinin is a promising drug for breast cancer but less favorable for chordoma therapy because a desirable anticancer drug induces apoptotic cell death in cancer cells at low doses with short time periods to be less cytotoxic to normal cells.

## Ethical Issues

 No ethical issues for this work.

## Conflict of Interest

 The authors declare no conflicts of interest.
